# Greater body mass index is a better predictor of subclinical cardiac damage at long-term follow-up in men than is insulin sensitivity: a prospective, population-based cohort study

**DOI:** 10.1186/s12872-015-0165-3

**Published:** 2015-12-10

**Authors:** Mette Lundgren Nielsen, Manan Pareek, Oke Gerke, Margrét Leósdóttir, Peter M. Nilsson, Michael Hecht Olsen

**Affiliations:** Cardiovascular and Metabolic Preventive Clinic, Department of Endocrinology, Centre for Individualized Medicine in Arterial Diseases (CIMA), Odense University Hospital, Sdr. Boulevard 29, DK-5000 Odense, Denmark; Department of Nuclear Medicine, Odense University Hospital, Odense and Centre of Health Economics Research, University of Southern Denmark, Odense, Denmark; Department of Cardiology, Skåne University Hospital, Malmö, Sweden; Department of Clinical Sciences, Lund University, Skåne University Hospital, Malmö, Sweden; Hypertension in Africa Research Team (HART), North-West University, Potchefstroom, South Africa

**Keywords:** Diastolic dysfunction, Homeostatic model assessment, Insulin sensitivity, Body mass index, Left ventricular mass, Prospective cohort study

## Abstract

**Background:**

To examine whether lower insulin sensitivity as determined by homeostatic model assessment (HOMA-%S) was associated with increased left ventricular mass (LVM) and presence of LV diastolic dysfunction at long-term follow-up, independently of body mass index (BMI), in middle-aged, otherwise healthy males.

**Methods:**

Prospective population-based cohort study with a median (IQR) follow-up time of 28 (27–28) years, in which traditional cardiovascular risk factors, including HOMA-%S and BMI, were assessed at baseline, and echocardiographic determination of LVM and LV diastolic function was performed at follow-up. Associations between risk factors and echocardiographic variables were tested using multivariable linear and binary logistic regression.

**Results:**

The study population comprised 247 men with a median (IQR) age of 47 (47–48) years. Mean (SD) BMI was 25.1 +/− 3.0 kg/m^2^, and median (IQR) HOMA-%S was 113.0 (68.3–284.6). Subjects with low insulin sensitivity (lowest HOMA-%S quartile (Q1)) had significantly greater BMI, fasting plasma insulin, and higher fasting blood glucose (FBG) (*p* <0.02 for all). BMI and HOMA-%S were significantly correlated (*r* = −0.383, *p* <0.0001). At follow-up, mean (SD) LVM and LVMI were 202 +/− 61 g and 103 +/− 31 g/m^2^, respectively, whereas median (IQR) E/é was 10 (8–12). Moreover, 36 % had grade 2 or 3 diastolic dysfunction. In multivariable analyses, greater BMI, but not low insulin sensitivity was independently associated with later detection of increased LVM and diastolic dysfunction.

**Conclusion:**

Greater baseline BMI, but not lower insulin sensitivity was independently associated with greater LVM and diastolic dysfunction at long-term follow-up.

**Electronic supplementary material:**

The online version of this article (doi:10.1186/s12872-015-0165-3) contains supplementary material, which is available to authorized users.

## Background

Diastolic dysfunction of the left ventricle (LV) is characterized by delayed active relaxation and increased chamber stiffness [1]. The condition is most commonly associated with ischemic heart disease and/or hypertension with subsequent concentric remodeling or hypertrophy of LV (LVH) [2]. LV diastolic dysfunction and LVH are powerful independent predictors of future cardiovascular morbidity and mortality [3, 4], and identification of other hemodynamic and non-hemodynamic factors associated with the development of these often subclinical cardiac conditions may unveil novel targets for prevention.

Both LV diastolic dysfunction and LVH are common findings among patients with diabetes mellitus (DM) [5–7]. The associations are independent of concomitant hypertension and ischemic heart disease, which has led to the term *diabetic cardiomyopathy*, defined as ventricular dysfunction in a patient with DM, occurring independently of an otherwise recognized cause [8]. However, the pathogenic mechanisms for development of this condition are poorly understood. Insulin resistance and the accompanying hyperinsulinemia may constitute an important pathophysiological link in subjects with obesity, glucose intolerance, or overt DM, but the majority of studies so far have yielded inconsistent results, particularly due to inadequate adjustment for key confounders, notably body size, blood pressure, and glucose levels [9–11].

Additionally, most previous studies addressing this subject have been of cross-sectional nature, which, besides the inability to establish causality, are further limited by the fact that there is evidence to suggest that the changes in LV structure and function may be consequences of long-term, rather than short-term, metabolic abnormalities [12].

Therefore, we conducted this study in subjects free from DM and overt cardiovascular disease at baseline aiming to examine whether lower insulin sensitivity as determined by homeostatic model assessment (HOMA-%S) was associated with increased left ventricular mass (LVM) and presence of LV diastolic dysfunction at long-term follow-up, independently of body mass index (BMI).

## Methods

### Study population

Study subjects were derived from the Malmö Preventive Project (MPP, 1974–1992, *n* = 33,346), a population-based cohort study aiming to screen for cardiovascular risk factors, alcohol abuse, and breast cancer among inhabitants in Malmö, Sweden, born between 1921 and 1949 [13]. All subjects answered a self-administered questionnaire on lifestyle, medical history, and current medication. Height and weight in light indoor clothing were measured, and body mass index (BMI) was calculated. Blood pressure was measured twice after 10 min of supine rest, with the mean value recorded for analysis. Moreover, blood samples were obtained after an overnight fast with measurement of blood glucose, plasma insulin, serum lipids, and serum creatinine. In 18,960 participants without prevalent DM, a 120 min oral glucose tolerance test (OGTT) was performed by a standard method (30 g/m^2^ body surface area (BSA) in a 10 % aqueous solution) [14]. A re-examination study (MPP-RES, *n* = 18,238) was conducted between 2002 and 2006. In a subsample of 1,792 individuals therefrom, an echocardiography and a 12-lead ECG recording were carried out. These subjects were randomly selected from groups defined by fasting plasma glucose (FPG), with oversampling in groups of subjects with impaired fasting glucose and DM, in order to ensure a sufficient number of individuals in each category. MPP and MPP-RES were approved by the Ethics Committee of Lund University, Sweden and conducted in accordance with the Declaration of Helsinki. Written informed consent was obtained from all participants.

#### Final study population

**Fig. 1:**
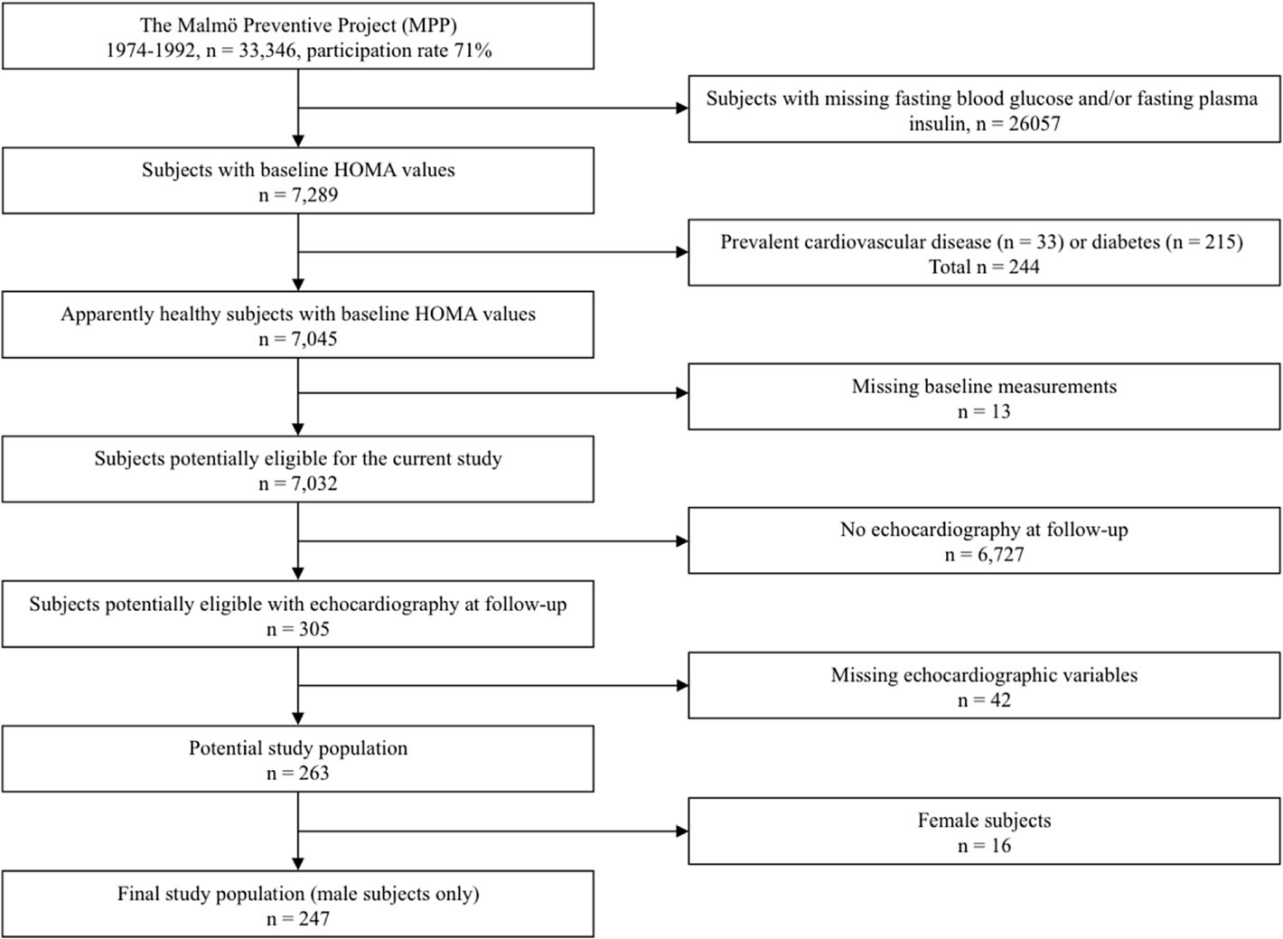
Flowchart showing the study population selection

Subjects with missing fasting blood glucose (FBG) and/or fasting plasma insulin measurements at baseline (*n* = 26,057) were excluded from the present study. Remaining subjects with prevalent cardiovascular disease (*n* = 33), DM (*n* = 215), and/or other missing baseline variables (*n* = 13), were likewise excluded. Of the 7032 subjects left, 305 subjects had an echocardiography performed at follow-up, with 263 subjects potentially eligible for the current study (missing echocardiography variables, *n* = 42). Since only 16 subjects were female, they were excluded as well, leaving a final study population comprising 247 males (Fig. 1). Relevant definitions are provided below.

### Investigations performed at baseline

#### Prevalent cardiovascular disease or diabetes mellitus

Prevalent cardiovascular disease was defined by the *International Classification of Diseases* (ICD-9 and ICD-10) codes gathered from the Swedish Hospital Discharge Registry as well as local hospital and study registries and encompassed previous myocardial infarction, transient ischemic attack, and stroke. Prevalent diabetes mellitus was defined as self-reported diabetes mellitus or according to the 1985 *World Health Organization (WHO)* criteria for DM by either FBG or during 120 min OGTT [15].

#### Insulin sensitivity (HOMA-%S)

HOMA-%S was derived via the computerized HOMA-calculator (©The University of Oxford 2004) using FBG and fasting plasma insulin (measured by standard radioimmunoassay) as input [16]. Based on sensitivity analyses regarding LV diastolic dysfunction, low insulin sensitivity was defined as the lowest HOMA-%S quartile (Q1), whereas quartiles 2–4 (Q2-4) were defined as normal insulin sensitivity.

### Investigations performed at follow-up

#### Echocardiography

Echocardiography was conducted with a 3V2c transducer (Acuson Sequoia, Mountain View, CA) or an S3 transducer (Sonos 5500 Philips, Andover, MA). LV ejection fraction (LVEF) was quantified visually. LVM calculations were based on 2-dimensional linear measurements in the parasternal long-axis view at the tips of the mitral valve leaflets at end-diastole, perpendicular to the long axis of LV. The thickness of the interventricular septum (IVS), LV internal diameter (LVID), and the thickness of the posterior wall (PW) were obtained by placing the calipers on the interface between myocardial wall and cavity and the interface between myocardial wall and pericardium, respectively. LVM was then calculated using the Cube formula recommended by the *American Society of Echocardiography (ASE)* and the *European Association of Cardiovascular Imaging (EACVI)*, and indexed for BSA, obtaining LVMI [17]*.* LV diastolic function was assessed in the apical four-chamber view using transmitral pulsed Doppler flow with a 1–3 mm sample volume placed between the tips of the mitral valve leaflets (obtaining E, A, and E-wave deceleration time (DT)) and tissue Doppler imaging with the sample volume positioned within 1 cm of the septal and lateral borders of the mitral annulus (obtaining both septal and lateral é and averaging the values for the analyses). A mean of 3–5 cycles was used. The intra- and interobserver variabilities are reported elsewhere [18]. Diastolic function was graded according to the recommendations of *American Society of Echocardiography* and *European Association of Cardiovascular Imaging* [19], using age-appropriate cut-off values of septal é, lateral é, E-wave DT, E/A, and averaged E/é. If septal é was ≥8 and/or lateral é was ≥10, subjects were classified as having normal diastolic function. If septal é was <8 and lateral é was <10, subjects were classified as having diastolic dysfunction, and the values of E-wave DT, E/A, and E/é were used for grading subjects into grade 1, 2 or 3 diastolic dysfunction, as previously described (Table 1) [20]. Equivocal cases, i.e. subjects who were in a transitional state between grade 1 and 2 diastolic dysfunction with E/é ≥9 and ≤12, but E/A and E-wave DT pointing in opposite directions, were classified as undetermined diastolic dysfunction. If E/é was >12, subjects were classified as having either grade 2 or 3 diastolic dysfunction. Finally, all subjects with E/é <9 were classified as either normal (E-wave DT <240 ms and E/A ≥0.8) or grade 1 diastolic dysfunction (all other subjects), even if they did not strictly fulfill the primary é criteria for normal diastolic function. Grade 2 and 3 diastolic dysfunction were grouped together, since only two individuals fulfilled the criteria for grade 3 diastolic dysfunction.

**Table 1:** Scheme for grading diastolic dysfunction

	Grade 0 (normal)	Grade 1 (mild)	Grade 2 (moderate)	Grade 3 (severe)
Septal é (cm/s)	≥8	<8	<8	<8
Lateral é (cm/s)	≥10	<10	<10	<10
E-wave DT (ms)	140–240	≥240	140–240	<140
E/A	0.8–1.5	<0.8	0.8–1.5	>1.5
E/é	<9	≤12	≥9	≥13

### Statistical analysis

Continuous variables were summarized by means and standard deviations (approximately normally distributed variables) and medians and interquartile ranges (IQR) (non-normally distributed variables), whereas categorical variables were presented by frequencies and corresponding percentages. Group-wise comparisons were performed using independent samples t-test, Mann-Whitney U-test, and Pearson’s χ^2^-test or Fisher’s exact test (depending on cell frequencies), respectively. The associations between risk factors and LVM, LVMI, or E/é, respectively, were assessed by multivariable linear regression. Since E/é was moderately positively skewed, the association between E/é and various risk factors was assessed by linear regression after natural log-transformation of E/é. The association between risk factors and diastolic function, i.e. normal or grade 1 diastolic dysfunction vs. grade 2 or 3 diastolic dysfunction, was assessed by binary logistic regression. Statistically and clinically significant variables were included in the final multivariable linear and binary logistic regression models, and stepwise subset selection was applied for adjustment of these models with a p-stay of 0.2. The significance level for the univariable analyses was 5 %. In all cases, time elapsed from baseline inclusion to echocardiography at follow-up was included as an explanatory variable under the assumption that it could impact the severity of the echocardiographic findings. All analyses were carried out using IBM SPSS Statistics 22 (IBM, Armonk, New York, USA) and Stata/IC 13 (StataCorp LP, College Station, Texas, USA).

## Results

### Characteristics of the study population

After applying the aforementioned exclusion criteria, a study cohort of 247 subjects was left for analysis. At baseline, subjects were middle-aged with a median age of 47 (IQR 47–48) years, mean systolic blood pressure 129 +/− 15 mmHg, and borderline overweight with mean BMI 25.1 +/− 3.0 kg/m^2^. Total cholesterol was mildly elevated at 5.7 +/− 1.0 mmol/L. Median HOMA-%S was 113.0 (IQR 68.3–284.6). Subjects with low insulin sensitivity (HOMA-%S Q1) had significantly greater BMI, fasting plasma insulin and both higher FBG and BG at 120 min, borderline significantly greater systolic blood pressure, but there were no significant between-group differences regarding age, smoking status, and total cholesterol. BMI and HOMA-%S were significantly correlated (Pearson’s *r* = −0.383, *p* <0.0001). At follow-up, mean LVMI was 103 +/− 31 g/m^2^, and median E/é was 10 (IQR 8–12). Moreover, 36 % (when excluding the 20 subjects with undetermined diastolic dysfunction) had grade 2 or 3 diastolic dysfunction. Subjects with low insulin sensitivity had a borderline significantly greater prevalence of grade 2 or 3 diastolic dysfunction, whereas there was no significant between-group difference with respect to LVEF. Tables 2 and 3 show the baseline characteristics of the subjects categorized according to HOMA-%S category and diastolic function, respectively. Furthermore, Additional file 1: Table S1 shows the baseline characteristics according to BMI category, i.e. non-overweight vs. overweight or obese subjects.

**Table 2:** Baseline characteristics according to HOMA-S category. Similar variables, obtained at follow-up, are depicted for comparison

Variable	All subjects (*n* = 247)	HOMA-S Q1 (*n* = 63)	HOMA-S Q2-4 (*n* = 184)	*P*-value for difference between HOMA-S categories
Baseline MPP				
Age (years)	47 [47–48]	47 [47–48]	47 [47–48]	0.1^c^
Active smoking	118 (48 %)	27 (43 %)	91 (50 %)	0.4^a^
BMI (kg/m^2^)	25.1 +/− 3.0	26.9 +/− 3.8	24.5 +/− 2.4	<0.0001^b^
Systolic blood pressure (mmHg)	129 +/− 15	133 +/− 19	128 +/− 14	0.0502^b^
Total cholesterol (mmol/L)	5.7 +/− 1.0	5.9 +/− 1.3	5.7 +/− 0.9	0.2^b^
Creatinine (μmol/L)	93 +/− 13	93 +/− 13	92 +/− 13	0.7^b^
FBG (mmol/L)	5.1 +/− 0.5	5.2 +/− 0.6	5.0 +/− 0.5	0.02^b^
Fasting insulin (pmol/L)	48 [18–78]	108 [90–144]	36 [18–54]	<0.0001^c^
HOMA-%B	83.2 [54.4–124.2]	147.2 [118.8–184.4]	67.3 [47.8–92.1]	<0.0001^c^
HOMA-%S	113.0 [68.3–284.6]	50.2 [37.1–61.9]	150.7 [99.3–289.7]	<0.0001^c^
Sedentary lifestyle	143 (58 %)	38 (60 %)	105 (57 %)	0.7^a^
Antihypertensive medication	11 (5 %)	4 (6 %)	7 (4 %)	0.4^a^
MPP re-examination				
Age (years)	74 [70–75]	74 [70–75]	74 [71–75]	0.5^c^
Active smoking	33 (13 %)	8 (13 %)	25 (14 %)	0.9^a^
BMI (kg/m^2^)	27.9 +/− 3.6	28.3 +/− 4.3	27.8 +/− 3.3	0.3^b^
Systolic blood pressure (mmHg)	149 +/− 21	146 +/− 18	149 +/− 21	0.3^b^
Total cholesterol (mmol/L)	5.1 +/− 1.1	4.7 +/− 1.0	5.2 +/− 1.1	0.001^b^
FPG (mmol/L)	7.1 +/− 2.1	7.4 +/− 2.0	7.0 +/− 2.1	0.1^b^
LVM (g)	202 +/− 61	205 +/− 61	201 +/− 61	0.7^b^
LVMI (g/m^2^)	103 +/− 30	103 +/− 30	103 +/− 30	0.9^b^
EF (%)	60 +/− 8	60 +/− 8	60 +/− 8	0.5^b^
Grade 2 or 3 diastolic dysfunction	82 (36 %)	27 (47 %)	55 (33 %)	0.06^a^
E/é	10 [8–12]	10 [8–12]	10 [7–12]	0.3^c^
Time (years)	28 [27–28]	28 [25–29]	28 [27–28]	0.4^c^

Categorical variables (active smoking, grade 2 or 3 diastolic dysfunction) are given as n (%), whereas continuous variables are given as mean +/− SD (approximately normally distributed variables, i.e. body mass index (BMI), systolic blood pressure, total cholesterol, creatinine, fasting blood glucose (FBG), fasting plasma glucose (FPG), left ventricular mass (LVM), left ventricular mass index (LVMI), ejection fraction (EF)) or median (IQR) (non-normally distributed variables, i.e. age, fasting insulin, HOMA-%S, HOMA-%B, E/é, and time)

^a^Pearson’s χ^2^-test; ^b^independent samples t-test; ^c^Mann-Whitney U test

**Table 3:** Baseline characteristics according to diastolic function. Similar variables, obtained at follow-up, are depicted for comparison

Variable	All subjects (*n* = 227)	Normal or grade 1 diastolic dysfunction (*n* = 145)	Grade 2 or 3 diastolic dysfunction (*n* = 82)	*P*-value for difference between diastolic dysfunction categories
MPP baseline				
Age (years)	47 [47–48]	47 [47–48]	47 [47–48]	0.5^c^
Active smoking	109 (48 %)	73 (50 %)	36 (44 %)	0.4^a^
BMI (kg/m^2^)	25.1 +/− 3.1	24.7 +/− 3.0	25.9 +/− 3.1	0.003^b^
Systolic blood pressure (mmHg)	129 +/− 15	129 +/− 16	129 +/− 15	0.7^b^
Total cholesterol (mmol/L)	5.7 +/− 1.0	5.7 +/− 0.9	5.7 +/− 1.2	0.8^b^
Creatinine (μmol/L)	92 +/− 13	91 +/− 12	94 +/− 14	0.09^b^
FBG (mmol/L)	5.1 +/− 0.5	5.1 +/− 0.5	5.1 +/− 0.5	0.6^b^
Fasting insulin (pmol/L)	48 [38–84]	42 [18–72]	54 [18–84]	0.1^c^
HOMA-%B	100.3 [68.7–137.9]	77.7 [52.0–117.6]	92.5 [58.0–133.6]	0.2^c^
HOMA-%S	111.0 [64.1–138.4]	124.4 [75.9–286.5]	99.6 [62.8–279.7]	0.1^c^
Sedentary lifestyle	133 (59 %)	86 (59 %)	47 (57 %)	0.8^a^
Antihypertensive medication	10 (4 %)	6 (4 %)	4 (5 %)	0.8^a^
MPP re-examination				
Age (years)	74 [70–75]	74 [71–75]	74 [70–75]	0.5^c^
Active smoking	32 (14 %)	22 (15 %)	10 (12 %)	0.5^a^
BMI (kg/m^2^)	27.9 +/− 3.5	27.4 +/− 3.3	28.9 +/− 3.7	0.002^b^
Systolic blood pressure (mmHg)	148 +/− 20	147 +/− 20	151 +/− 21	0.2^b^
Total cholesterol (mmol/L)	5.1 +/− 1.1	5.1 +/− 1.1	4.9 +/− 1.0	0.2^b^
FPG (mmol/L)	7.1 +/− 2.1	7.0 +/− 2.0	7.2 +/− 2.2	0.5^b^
LVM (g)	202 +/− 63	197 +/− 64	212 +/− 58	0.1^b^
LVMI (g/m^2^)	103 +/− 31	101 +/− 32	106 +/− 28	0.2^b^
EF (%)	60 +/− 9	60 +/− 8	62 +/− 10	0.09^b^
E/é	10 [7–12]	8 [7–10]	13 [12–15]	<0.0001^c^
Time (years)	28 [28–29]	28 [27–29]	28 [25–28]	0.02^c^

Categorical variables (active smoking) are given as n (%), whereas continuous variables are given as mean +/− SD (approximately normally distributed variables, i.e. body mass index (BMI), systolic blood pressure, total cholesterol, creatinine, fasting blood glucose (FBG), fasting plasma glucose (FPG), left ventricular mass (LVM), left ventricular mass index (LVMI), ejection fraction (EF)) or median (IQR) (non-normally distributed variables, i.e. age, insulin, HOMA-%S, HOMA-%B, E/é, and time)

^a^Pearson’s χ^2^-test; ^b^independent samples t-test; ^c^Mann-Whitney U test

### Left ventricular size

There were no significant differences in neither LVM (205 +/− 61 g/m^2^ vs. 201 +/− 61 g/m^2^, *p* = 0.7) nor LVMI (103 +/− 30 g/m^2^ vs. 103 +/− 30 g/m^2^, *p* = 0.9) between subjects with low vs. normal insulin sensitivity according to HOMA-%S. In univariable analyses, higher values of both LVM and LVMI were significantly associated with higher BMI, but not HOMA-%S category. The adjusted multivariable linear regression models are presented in Tables 4 and 5 and included only BMI, whereas age and HOMA-%S category were forced into the models. Smoking status, systolic blood pressure, total cholesterol, fasting plasma insulin, creatinine, and sedentary lifestyle were not significantly associated with LVM or LVMI on univariable analyses and therefore not included in the final multivariable regression models. We did not detect any significant interactions regarding HOMA-%S. Furthermore, the results were not affected by BMI alterations during follow-up, i.e. whether the individuals gained or lost weight, even when stratified for whether or not they where initially overweight (results not shown, available upon request).

**Table 4:** Multivariable linear regression model for the prediction of LVM at follow-up (adjusted r^2^ = 0.088)

Risk factor	*β*-coefficient (95 % CI)	*P*-value
Age (per year)	0.10 (−1.84 to 2.05)	0.9
BMI (per kg/m^2^)	6.69 (4.05 to 9.33)	<0.0001
HOMA-%S Q1 vs. Q2-4	−12.46 (−30.19 to 5.27)	0.17

**Table 5:** Multivariable linear regression model for the prediction of LVMI at follow-up (adjusted r^2^ = 0.040)

Risk factor	*β*-coefficient (95 % CI)	*P*-value
Age (per year)	0.37 (−0.61 to 1.36)	0.5
BMI (per kg/m^2^)	2.24 (0.91 to 3.58)	0.001
HOMA-%S Q1 vs. Q2-4	−5.18 (−14.17 to 3.799)	0.3

### Diastolic function

There was no significant difference in E/é according to HOMA-%S category (Q1: median 10 (IQR: 8–12) vs. Q2-4 median: 10 (IQR: 7–12), *p* = 0.2)), whereas grade 2 or 3 diastolic dysfunction was borderline significantly more prevalent among subjects with low vs. normal HOMA-%S (47 % vs. 33 %, *p* = 0.06). In univariable analyses, higher E/é was associated with higher age, BMI, serum creatinine, and shorter follow-up time, while diastolic dysfunction was associated with higher BMI and shorter follow-up time, but not serum creatinine. The adjusted multivariable regression models are shown in Tables 6 and 7. In both cases, HOMA-%S was forced into the models. Smoking status, systolic blood pressure, total cholesterol, fasting plasma insulin, and sedentary lifestyle were not significantly associated with E/é or the presence of diastolic dysfunction on univariable analyses and therefore not included in the multivariable regression models. No significant interactions were detected with respect to HOMA-%S. Regarding BMI changes, the same was true as for LV size (results not shown, available upon request).

**Table 6:** Multivariable linear regression model for the prediction of log(E/é) at follow-up (adjusted r^2^ = 0.152)

Risk factor	*β*-coefficient (95 % CI)	*P*-value
Age (per year)	0.02 (0.006 to 0.027)	0.003
BMI (per kg/m^2^)	0.03 (0.02 to 0.04)	<0.0001
Creatinine	0.002 (−0.001 to 0.005)	0.18
Time (per year)	−0.03 (−0.06 to −0.01)	0.01
HOMA-%S Q1 vs. Q2-4	−0.04 (−0.13 to 0.06)	0.5

**Table 7:** Binary logistic regression model for the prediction of grade 2 or 3 diastolic dysfunction at follow-up (Nagelkerke r^2^ = 0.078)

Risk factor	Odds ratio (95 % CI)	*P*-value
Age (per year)	1.03 (0.94 to 1.13)	0.5
BMI (per kg/m^2^)	1.12 (1.01 to 1.24)	0.03
Time (per year)	0.86 (0.73 to 1.02)	0.07
HOMA-%S Q1 vs. Q2-4	1.30 (0.66 to 2.56)	0.4

## Discussion

In this prospective population-based cohort study comprising middle-aged male, apparently healthy subjects, we found that greater BMI, but not lower insulin sensitivity defined as the lowest HOMA-%S quartile was associated with later detection of increased LVM and grade 2 or 3 LV diastolic dysfunction. Low insulin sensitivity (HOMA-%S Q1) was only associated later recognition of grade 2 or 3 LV diastolic dysfunction in univariable analysis, but the association was lost after adjusting for BMI. As expected, BMI and HOMA-%S were significantly correlated.

Conflicting results have been reported regarding the relationship between insulin sensitivity and both indexed and non-indexed LVM, and to our knowledge, none of these were prospective. In a small study comprising 29 non-obese, glucose-tolerant subjects with borderline hypertension, *Phillips* et al. found a significant independent association between LVMI and insulin sensitivity [21]. Subjects were age-wise comparable to our cohort; however, insulin sensitivity was derived by frequent sampling during OGTT. Likewise, *Sundström* et al. [22] found a borderline significant association between insulin resistance according to HOMA (HOMA-IR) and LVMI in normotensive subjects, but they did not adjust for glucometabolic status. On the contrary, in one of the largest studies to date, based on the *Framingham Heart Study* [10] cohort, HOMA-IR was associated with increased LVM among women only, although the relationship was largely accounted for by obesity. Further supporting our results, neither *Galvan* et al. [11] nor *Ebinc* et al. [23] were able to detect an association between LVM and insulin resistance, independently of BMI, in subjects without DM. Although a number of other studies exist, direct comparison is challenging, especially due to limited sample sizes and heterogeneous study populations, with the majority having assessed subjects already at increased cardiovascular risk, i.e. subjects who were obese, had DM, or were hypertensive. Such individuals have greater insulin resistance and LVM than the general population [24, 25], providing limited pathophysiological understanding regarding subjects in whom non-hemodynamic LVH-inducing mechanisms are likely to be more important. Adjustment for these important confounders has also been quite variable [11]; for instance, some of the described relations of insulin resistance to LVM may have been mainly due to the effects of blood pressure. A true association between insulin LV size and function is therefore more likely be revealed in a general population-based study.

Asymptomatic LV diastolic dysfunction is the most prominent characteristic of diabetic cardiomyopathy [8, 26]. However, since LV diastolic function may already be impaired in the pre-diabetic or even preclinical phase of glucometabolic disturbances, i.e. before the onset of sustained hyperglycemia, an independent mechanistic role of insulin resistance may exist [27–29]. Furthermore, LV diastolic dysfunction may be evident at less severe insulin resistance when compared to the values associated with measureable LV structural changes [30]. In a population-based study including 1063 subjects, *Fontes-Carvalho* et al. found a significant association between higher insulin resistance according to HOMA-IR and worse LV diastolic function, including lower lateral é velocity and higher E/é ratio [31]; however, without adjusting for systolic blood pressure and BMI. Further supporting these results, both *Dinh* et al. [32] and *Hwang* et al. [30] found an independent association between insulin resistance and both presence and severity of LV diastolic dysfunction, independently of overt DM; however, neither studies adjusted for body size. In addition, all the above mentioned studies were cross-sectional. In the present study, we showed that BMI itself, but not insulin sensitivity, was significantly associated with E/é and grade 2 or 3 diastolic dysfunction, in multivariable analysis. Nevertheless, direct comparison between different studies is complicated by use of variable methods for assessment of both LV diastolic function and insulin sensitivity as well as inadequate adjustment for relevant confounders.

The highly prevalent co-existence of insulin resistance, hyperinsulinemia, obesity, hypertension, and DM makes it difficult to dissect the separate role of each of these conditions for development of subclinical cardiac damage [11]. However, at present time, there is insufficient convincing evidence to conclude that LVM and prevalence of LV diastolic dysfunction are greater among subjects with low insulin sensitivity or insulin resistance, when adequate care is taken to adjust for DM, blood pressure, and body size. Therefore, uncertainty remains, as to why subjects with increased body size have greater LV size and worse diastolic function [33–35]. Several other risk factors, e.g. elevated blood pressure, glucose and cholesterol levels, are associated with obesity, and the progressive addition of metabolic risk factors seems to be associated with greater LVM [36]. Just as proposed for diabetic cardiomyopathy, the myocardial alterations associated with obesity are likely to be a result of several similar synergistically acting mechanisms [8], and it is possible that low insulin sensitivity, as previously suggested for fasting plasma glucose [20], primarily acts as an effect modifier of these other risk factors. This hypothesis is supported by the fact that most studies reporting a positive association between insulin resistance and subclinical cardiac damage have involved subjects with other risk factors as well, and our chances of finding positive associations were weakened by the strict selection criteria employed, aiming to exclude subjects with prevalent DM or cardiovascular disease. Although we were not directly able to find synergistic interactions between insulin sensitivity and the traditional risk factors in the present study, this could have resulted from the relatively small sample size. The exact reason why baseline BMI, but not insulin sensitivity, was associated with later cardiac damage in our study, is uncertain. However, HOMA-indices display pronounced biological variation, which decreases the chances of finding such significant associations. Furthermore, the HOMA-indices show considerable temporal changes, which may further reduce the predictive value at long-term follow-up. Although BMI changes over time as well, the fluctuations may more often be unidirectional [37, 38].

Regardless of whether or not insulin resistance is the main mediator of subclinical cardiac damage in obesity, pre-diabetes, and DM, the strong association between higher baseline BMI and later detection of structural and functional LV changes in our study suggests that early weight loss in overweight or obese subjects may halt the progression of adverse cardiac alterations, specifically reduce the risk of LVH and diastolic dysfunction. This is supported by the results from the *Coronary Artery Risk Development in Young Adults* study, in which increasing BMI over a 5- to 10-year follow-up in generally healthy adults was associated with increasing LVM [33, 34]. Further supporting this early preventive strategy is the weak ability of therapeutic interventions, e.g. intensive glycemic control [39, 40] and antihypertensive drugs [41, 42], to lower cardiovascular risk, when DM is overt, because the myocardial damage may have become partly to completely irreversible at this point. Lastly, early weight loss is associated with other clinically relevant benefits, including a lower risk of hypertension, dyslipidemia, and DM itself, which may further reduce the risk of cardiovascular complications [43]. The lack of our detection of a beneficial effect of BMI reduction in the present study was most likely related to the small number (*n* = 32) of subjects who actually lost weight during the study period and the possible BMI fluctuations over time that we were unable to account for. Even in those having lost weight, the loss was very subtle in most subjects. In addition, most individuals were normo- or overweight, but not obese, making detection of substantial benefits difficult.

### Limitations

Although the participation rates of 71 % in MPP and 72 % in MPP-RES, respectively, are considered high, one may still argue that the study subjects did not represent a truly random population sample since people who agree to take part may be healthier than the general population. All subjects in the present study were male, limiting the applicability of the results in females. Moreover, our exclusion of a vast amount of the original study population in order to get a cohort of apparently healthy subjects, who were alive and underwent echocardiography, however with variable follow-up periods, may introduce further selection bias, including survival bias, and lack of adequate power.

Insulin sensitivity was not assessed according to the gold standard method, i.e. the hyperinsulinemic euglycemic clamp technique [44]. However, the use of fasting BG and insulin was justified by the fact that HOMA-derived parameters are strongly related to clamp-measured insulin sensitivity and insulin resistance in subjects both with and without DM [27, 45]. Sensitivity analyses with respect to the prediction of LV size and LV diastolic dysfunction were performed, before settling on the use of Q1 as cut-off in the present study. Moreover, data on glucose tolerance would have been desirable; however, in the present study, inclusion of these data would have reduced sample size even further.

Similarly, the use of BMI as a surrogate marker for obesity has some limitations [46–48], and the inclusion of other measures, e.g. waist circumference and waist-to-hip ratio would have been preferred; however, these measurements were not available. The accuracy of BMI for diagnosing obesity is especially limited for individuals with BMI between 25.0 and 29.9 kg/m^2^, in men and in the elderly. However, BMI or plain body weight may still be the best way to evaluate changes in body fat content over time, because increments in body weight or BMI most likely represent fat gain [49].

Linear LV measurements have prognostic value and are feasible, especially when studying large populations. However, the method is based solely on basal dimensions, unable to accommodate for LV shape and size changes that might occur along the long axis of the chamber, and the formula for calculating LVM assumes normal LV geometry and cubes the linear measurements. Therefore, even small errors may significantly influence the calculated mass [17]. Although echocardiography does not directly measure the same parameters for diastolic function that are measured invasively, it is still the most practical and recommended routine clinical approach. However, some limitations deserve mentioning. Minimal angulation is essential for reliable spectral Doppler measurements. Additionally, the usefulness of é velocity in normal subjects may be limited, as preload increases é in these subjects. The correlation between E/é between 8 and 15 using septal é (9–13 in the present study) and mean LV diastolic pressure displays wide variability [50]. Therefore, although increased E/é is indicative of an elevated LV filling pressure, it should not be used as stand-alone parameter when drawing conclusions about LV diastolic dysfunction. Furthermore, our grading of LV diastolic dysfunction could have been more robust, had we also been able to incorporate the left atrial volume index [19]. Lastly, the lack of an echocardiography at baseline prevented us from directly assessing LV structural and functional changes over time.

## Conclusion

In conclusion, in a prospective population-based cohort study including apparently healthy middle-aged male subjects, greater baseline BMI, but not lower insulin sensitivity was independently associated with greater LVM and diastolic dysfunction at long-term follow-up.

### Ethics, consent and permissions

All subjects provided informed consent to participate in this study.

## Additional file

Additional file 1: Table S1. Baseline characteristics according to BMI category. (DOCX 17 kb)

## Abbreviations

BMI: Body mass index
BSA: Body surface area
DM: Diabetes mellitus
DT: E-wave deceleration time
ECG: Electrocardiography
FBG: Fasting blood glucose
FPG: Fasting plasma glucose
HOMA: Homeostatic model assessment
ICD: International classification of diseases
IQR: Interquartile range
LV: Left ventricular / left ventricle
LVEF: Left ventricular ejection fraction
LVH: Left ventricular hypertrophy
LVM: Left ventricular mass
LVMI: Left ventricular mass index
MPP: Malmö preventive project
MPP-RES: Malmö preventive project re-examination study
OGTT: Oral glucose tolerance test
WHO: World Health Organization
