# Supplementary material for: Greater body mass index is a better predictor of subclinical cardiac damage at long-term follow-up in men than is insulin sensitivity: a prospective, population-based cohort study
Source: BMC Cardiovasc Disord. 2015 Dec 10;15:168. doi: 10.1186/s12872-015-0165-3 (PMC4676144; doi:10.1186/s12872-015-0165-3)
Supplement: Additional file 1: Table S1. — Baseline characteristics according to BMI category. (DOCX 17 kb) [file 12872_2015_165_MOESM1_ESM.docx]

| **Variable** | **All subjects**  **(n = 247)** | **BMI < 25 kg/m^2^**  **(n = 131)** | **BMI >/= 25 kg/m^2^**  **(n = 116)** | **P-value for difference between BMI categories** |
| --- | --- | --- | --- | --- |
| **MPP baseline** |  |  |  |  |
| **Age (years)** | 47 [47-48] | 47 [47-48] | 47 [47-48] | 0.01 |
| **BMI (kg/m^2)^** | 25.1 +/- 3.0 | 22.9 +/- 1.4 | 27.6 +/- 2.3 | < 0.0001 |
| **Active smoking** | 118 (48 %) | 73 (56 %) | 45 (39 %) | 0.008 |
| **Systolic blood pressure (mmHg)** | 129 +/- 15 | 127 +/- 14 | 132 +/- 16 | 0.02 |
| **Total cholesterol (mmol/L)** | 5.7 +/- 1.0 | 5.6 +/- 1.0 | 5.9 +/- 1.1 | 0.0546 |
| **Creatinine (µmol/L)** | 93 +/- 13 | 91 +/- 13 | 94 +/- 13 | 0.1 |
| **FBG (mmol/L)** | 5.1 +/- 0.5 | 5.0 +/- 0.5 | 5.2 +/- 0.6 | 0.04 |
| **Fasting insulin (pmol/L)** | 48 [18-78] | 36 [18-60] | 60 [36-96] | < 0.0001 |
| **HOMA-%B** | 83.2 [54.4-124.2] | 69.3 [47.8-109.5] | 98.4 [66.2-138.9] | < 0.0001 |
| **HOMA-%S** | 113.0 [68.3-284.6] | 150.2 [88.4-291.1] | 87.0 [57.0-144.6] | < 0.0001 |
| **MPP re-examination** |  |  |  |  |
| **LVM (g)** | 202 +/- 61 | 187 +/- 53 | 219 +/- 64 | < 0.0001 |
| **LVMI (g/m^2^)** | 103 +/- 30 | 97 +/- 26 | 109 +/- 33 | 0.002 |
| **EF (%)** | 60 +/- 8 | 61 +/- 7 | 59 +/- 9 | 0.1 |
| **Grade 2 or 3 DD** | 82 (36 %) | 36 (30 %) | 46 (43 %) | 0.03 |
| **E/é** | 10 [8-12] | 9 [7-12] | 10 [8-13] | 0.001 |
| **Time (years)** | 28 [27-28] | 28 [26-29] | 28 [28-29] | 0.8 |

**Additional file 1: Table S1: Baseline characteristics according to BMI category.**

Categorical variables (active smoking, grade 2 or 3 diastolic dysfunction) are given as n (%), whereas continuous variables are given as mean +/- SD (approximately normally distributed variables, i.e. BMI, systolic blood pressure, total cholesterol, creatinine, FBG, LVM, LVMI, and EF) or median (IQR) (non-normally distributed variables, i.e. age, fasting insulin, HOMA-%S, HOMA-%B, E/é, and time).

*Pearson’s χ^2^-test; ^ϯ^independent samples t-test; ^§^Mann-Whitney U test.
